# Retrospectively gated intracardiac 4D flow MRI using spiral trajectories

**DOI:** 10.1002/mrm.25612

**Published:** 2015-02-13

**Authors:** Sven Petersson, Andreas Sigfridsson, Petter Dyverfeldt, Carl‐Johan Carlhäll, Tino Ebbers

**Affiliations:** ^1^ Division of Cardiovascular Medicine, Department of Medical and Health Sciences Linköping University Linköping Sweden; ^2^ Center for Medical Image Science and Visualization Linköping University Linköping Sweden; ^3^ Department of Clinical Physiology Karolinska Institutet and Karolinska University Hospital Stockholm Sweden; ^4^ Department of Clinical Physiology and Department of Medical and Health Sciences Linköping University Linköping Sweden; ^5^ Division of Media and Information Technology, Department of Science and Technology/Swedish e‐Science Research Centre Linköping University Linköping Sweden

**Keywords:** spiral, 4D flow; phase‐contrast MRI, magnetic resonance imaging, cardiac flow, retrospective cardiac gating

## Abstract

**Purpose:**

To develop and evaluate retrospectively gated spiral readout four‐dimensional (4D) flow MRI for intracardiac flow analysis.

**Methods:**

Retrospectively gated spiral 4D flow MRI was implemented on a 1.5‐tesla scanner. The spiral sequence was compared against conventional Cartesian 4D flow (SENSE [sensitivity encoding] 2) in seven healthy volunteers and three patients (only spiral). In addition to comparing flow values, linear regression was used to assess internal consistency of aortic versus pulmonary net volume flows and left ventricular inflow versus outflow using quantitative pathlines analysis.

**Results:**

Total scan time with spiral 4D flow was 44% ± 6% of the Cartesian counterpart (13 ± 3 vs. 31 ± 7 min). Aortic versus pulmonary flow correlated strongly for the spiral sequence (*P* < 0.05, slope = 1.03, R^2^ = 0.88, N = 10), whereas the linear relationship for the Cartesian sequence was not significant (*P* = 0.06, N = 7). Pathlines analysis indicated good data quality for the spiral (*P* < 0.05, slope = 1.02, R^2^ = 0.90, N = 10) and Cartesian sequence (*P* < 0.05, slope = 1.10, R^2^ = 0.93, N = 7). Spiral and Cartesian peak flow rate (*P* < 0.05, slope = 0.96, R^2^ = 0.72, N = 14), peak velocity (*P* < 0.05, slope = 1.00, R^2^ = 0.81, N = 14), and pathlines flow components (*P* < 0.05, slope = 1.04, R^2^ = 0.87, N = 28) correlated well.

**Conclusion:**

Retrospectively gated spiral 4D flow MRI permits more than two‐fold reduction in scan time compared to conventional Cartesian 4D flow MRI, while maintaining similar data quality. Magn Reson Med 75:196–206, 2016. © 2015 Wiley Periodicals, Inc.

## INTRODUCTION

Altered intracardiac blood flow has been recognized in the settings of various cardiac abnormalities, including distorted wall motion 1, valvular dysfunction 2, and arrhythmia 3, and is increasingly studied while methods for visualization and quantification have been developed and validated. Time‐resolved three‐dimensional (3D) phase‐contrast MRI, referred to as 4D flow MRI 4, 5, is a powerful tool for the visualization and quantification of blood flow and creates the opportunity to calculate a range of unique hemodynamic parameters.

The use of 4D flow MRI has increased the understanding of normal and abnormal cardiac blood flow 6, 7, 8, 9, 10, 11 and has proven valuable for various clinical applications 7, 12, 13, 14, 15, 16. However, cardiac applications of 4D flow MRI require large volumetric coverage, resulting in scan times of about 15 to 40 minutes, which may be prohibitive for some patients. Reduced scan time can be expected to expand the clinical and investigative use of 4D flow MRI.

Scan time reductions can be achieved by data undersampling or by the more efficient traversal of k‐space. The primary drawbacks of data undersampling are reduced signal‐to‐noise ratio (SNR) or temporal smoothing. The most commonly used methods for decreasing the scan time for cardiac flow imaging are parallel imaging 17, 18 and segmented k‐space sampling 19. Parallel imaging utilizes the coil sensitivity variations of multiple coil elements and can be used to reconstruct undersampled data without aliasing artifacts. Parallel imaging using SENSE (sensitivity encoding) together with partial Fourier techniques has been used successfully for retrospectively gated 4D flow MRI of the whole heart, obtaining a net acceleration factor of 4 (SENSE 3; 75% k‐space coverage) 20. Correlations in both k‐space and time can be used to reduce the number of samples needed 21, 22, 23. Spatiotemporal parallel imaging methods such as k‐t SENSE, k‐t BLAST (broad‐use linear speed‐up technique), and k‐t GRAPPA (generalized autocalibrating partially parallel acquisitions) have been used to reduce scan times for Cartesian phase‐contrast MRI, resulting in a net acceleration of around 5 24, 25, 26. However, some temporal smoothing remains for k‐t approaches. Principal component analysis 27 seems promising to reduce the scan time with acceleration factors up to 8 for 4D flow 28, but it has not yet been demonstrated for cardiac 4D flow. Moreover, compressed sensing in combination with Poisson‐disk undersampling has been used to accelerate 4D flow MRI measurements with accelerations factors of 2 to 6 29, 30.

An alternative approach to decreasing scan time is to sample k‐space more efficiently. This can be done by traversing the k‐space more quickly, that is, sampling the k‐space with a higher bandwidth and stronger gradients. A higher bandwidth is not cost‐effective in conventional Cartesian or radial imaging because of the time required for excitation and velocity encoding. Radial sampling has been used for 4D flow imaging of the heart and vessels 31, 32, 33 and is suitable for improved spatial resolution. This technique has several advantages, including great flexibility in reconstruction and self‐gating. A reduction in scan time for radial imaging can be achieved in combination with undersampling, which leads to a loss of SNR and streak aliasing artifacts. When compared to radial trajectories, spiral trajectories can cover a larger part of k‐space during a readout; therefore, they can be used to reduce scan times without sacrificing SNR. Increased bandwidth allows for further reductions in scan time. The use of spiral acquisition has been hindered by sensitivity to system imperfections and off‐resonance caused by chemical shifts or main field inhomogeneities. Moreover, the image reconstruction is more time consuming and computationally demanding compared to a Cartesian acquisition. Recent advances in hardware and computational algorithms may allow corrections that limit these effects. Spiral MRI has previously been used for 2D flow imaging 34, 35, 36, 3D MR angiography 37, 38, 39, and 2D myocardial phase velocity mapping 40. In addition to reducing the scan time, spiral k‐space trajectories also are inherently insensitive to flow‐induced displacement 41, 42. Moreover, because spiral k–space trajectories start in the center of k‐space, they may be suitable for respiratory self‐gating.

Recently, spiral readouts have also been used successfully for 4D flow MRI in the carotid arteries and in the aorta using prospective cardiac gating 43, 44. However, when using prospective cardiac gating, typically only about 80% to 90% of the cardiac cycle is covered, missing the majority of blood flow during atrial contraction at the end of diastole 45. This phase of diastolic filling is important from both a physiological and methodological point of view. Although late diastolic flow is less important for volume flow assessment in the large arteries, it is essential in the assessment of venal and cardiac blood flow. The early‐/late‐transmitral inflow velocity ratio is a widely used clinical descriptor of left ventricle (LV) diastolic function, which can only be assessed based on data representing the entire diastole. In addition, the assessment of flow in the cardiac atria can only be conclusive if data are acquired throughout the entire cardiac cycle. Moreover, tracing of pathlines from diastole to systole has shown to be a powerful approach for both qualitative and quantitative intracardiac blood flow analysis 46. In order to cover the entire cardiac cycle, retrospective cardiac gating is required.

The aim of this work is to develop a retrospectively cardiac‐gated 4D flow MRI sequence using a stack of spiral readouts and to evaluate it for volume flow and pathline analysis of intracardiac blood flow over the complete cardiac cycle.

## METHODS

A retrospectively electrocardiogram‐gated 3D three‐directional velocity imaging pulse sequence using stacks of spiral readouts was implemented on a clinical 1.5‐tesla (T) MR scanner. The performance of this spiral 4D flow MRI sequence was evaluated in‐vivo against a conventional Cartesian 4D flow acquisition by comparison of the net volume flow in the ascending aorta and the pulmonary artery. Because 4D flow data are often used for particle trace analysis, a quantitative pathline‐based evaluation was performed on the conventional and spiral 4D flow MRI acquisitions.

### In‐Vivo Measurements

A clinical 1.5‐T MR system (Achieva; Philips, Best, The Netherlands) with 33 mT/m gradient strength and 180 T/m/s slew rate and a five‐element cardiac SENSE coil was used for all acquisitions. A stack of spirals was used to traverse a 3D k‐space, and every slice encoding partition consisted of 10 spiral interleaves. For every slice encoding, all 10 interleaves were acquired before acquiring the next slice encoding, starting in the center of k‐space. Because signal from fat may cause blurring in spiral images due to off‐resonance, a 1‐1 spectral–spatial excitation pulse was used in the spiral acquisition to suppress the signal from fat 44. Furthermore, in order to reduce off‐resonance artifacts and preserve an adequate temporal resolution, the length of the spiral readouts was limited to 5 ms. The standard variable density spiral readouts implemented on the scanner were used.

Ten subjects, consisting of seven healthy volunteers (3 women and 4 men, age 27 ± 3 years, heart rate 63 ± 10 beats per minute) and three patients were imaged after approval by the regional ethics committee and with written consent from all participants. The patients were referred to the cardiac MRI unit for clinical examination. All patients were in sinus rhythm and had a heart rate of 70 ± 16 beats per minute (patient 1: 52‐year‐old female with ischemic heart disease and a mildly dilated LV with moderate systolic dysfunction; patient 2: 19‐year‐old female with intermittent arrhythmia and a LV with normal size and normal systolic function; patient 3: 30‐year‐old male with repaired congenital heart disease [Senning repair for transposition of the great arteries] and a moderately dilated right [systemic] ventricle with mild systolic dysfunction).

Spiral 4D flow data were obtained for all participants. Cartesian data were collected only for the healthy volunteers because the total scan time for the two 4D flow measurements was considered too long for the patients. Retrospective cardiac gating was used in all flow measurements. For respiratory gating of the 4D flow measurements, a navigator with an acceptance window of 4 mm in the central 25% of k‐space and 7 mm in the outer parts was used. The acquisition parameters for all flow measurements are listed in Table 1. In the spiral sequence, three velocity encoded segments and one reference segment for a single line in k‐space were acquired in succession within the same heart phase, resulting in a nominal temporal resolution of 4 × TR. Due to the shorter TR of the Cartesian sequence, two k‐space lines could be collected for every heart phase using the Cartesian sequence, resulting in about the same nominal temporal resolution calculated as 2 × 4 × TR.

**Table 1:** Flow Measurement Parameters

	Spiral 4D	Cartesian 4D
Echo time/TR	3.7/12 ms	3.4/5.8 ms
Number of interleaves	10	–
Readout duration	5 ms	2.5 ms
SENSE factor	1	2
Segmentation factor	1	2
FOV	280 × 280 mm^2^	235–280 × 280 mm^2^
Matrix size	100 × 100	84–100 × 100
Slices*	36–40	36–40
Voxel size	2.8‐mm isotropic	2.8‐mm isotropic
Temporal resolution	48–48.8 ms	46.4–46.5 ms
Flip angle	8°	6.5°
Velocity encoding	120 cm/s	120 cm/s
Nominal scan time	460–510 RR intervals	1012–1326 RR intervals
Actual scan time (without patients, mean + SD)	13 ± 3 min	31 ± 7 min

*Reconstructed slices. A slice oversampling factor of 27% was used to avoid foldover.

FOV = field of view; SENSE, sensitivity encoding; SD, standard deviation; TR, repetition time; RR, R‐wave to R‐wave.

In addition to the velocity measurements, morphological cine‐balanced steady‐state free‐precession (bSSFP) imaging was used to acquire three‐ and four‐chamber‐long axis images and a stack of short‐axis images during end‐expiratory breath holds. The bSSFP data were reconstructed to 30 time frames. The following parameters were used for the long‐axis bSSFP images: repetition time (TR)/echo time (TE) 3.3/1.63 ms, voxel size 1.67 × 1.67 × 8 mm^3^, field of view (FOV) 320 × 320 mm^2^, one breath hold. The following parameters were used for the short‐axis bSSFP images: TR/TE 3.3/1.63 ms, voxel size 2 × 2 × 6 mm^3^, FOV 350 × 350 mm^2^, 18 slices, four to five breatholds. The slice thickness was 8 mm, and the acquired pixel size was 1.67 × 1.67 mm^2^ and 2 × 2 mm^2^ for the long‐ and short‐axis images, respectively. The stack of short‐axis images consisted of 18 slices of 6 mm. The bSSFP images were collected before the flow measurements. An extra short‐axis acquisition was obtained before the second flow scan to determine possible patient movement. The best‐aligned short‐axis stack was used for segmentation. The 4D‐flow scans were carried out in a randomized order.

### In‐Vitro Measurements

In‐vitro measurements on a stationary phantom were performed in order to compare the SNR and velocity offsets of the spiral and Cartesian 4D‐flow measurements. Only a single timeframe was acquired, and no respiratory motion or cardiac gating was simulated. Other than that, the 4D‐flow sequences were identical to the in‐vivo measurements. Additional noise images were measured with all gradients and radiofrequency pulses turned off.

### Postprocessing

The Cartesian data were reconstructed on the scanner, whereas the spiral data were reconstructed using ReconFrame (Gyrotools Inc., Zurich, Switzerland). Using retrospective cardiac gating, the acquired k‐space trajectories can be combined into an arbitrary number of fully sampled k‐space datasets. In this work, 40 time frames were reconstructed for all flow data, which ensures that all temporal variations present in the acquired data are also reflected in the reconstructed data. This was accomplished by normalizing all cardiac cycles to one average cardiac cycle. Because most of the variation of heart rate occurs in diastole, the data was sorted differently for systole and diastole, keeping systole constant and stretching diastole.

The effects of concomitant gradient terms were automatically corrected for in the reconstruction 47. Phase offsets due to eddy currents were corrected by subtraction of a fourth‐order polynomial fitted to static tissue detected by low temporal variance 48. Initial tests indicated that a fourth‐order polynomial provided the best correction for these sequences and applications.

### Data Analysis

The net volume flow rate per cardiac cycle from the 4D flow measurements was computed in the proximal ascending aorta just downstream of the sinotubular junction and pulmonary trunk between the pulmonary valve and bifurcation. After segmentation of the vessels, using a semiautomatic algorithm, the net volume flow rate per cardiac cycle was computed for all flow measurements. Moreover, the peak flow rate and peak velocity in these segmentations were calculated.

4D flow MRI is often used for visualization of blood flow patterns using particle trace visualization. Thus, further evaluation of the 4D flow data was performed using quantitative pathline analysis 46. Pathlines are very sensitive to errors in the velocity measurements because pathlines are integrated over time and errors accumulate over the course of the trajectory. By releasing pathlines backward and forward from a segmentation of the LV at the end diastole and using segmentation at the end systole to determine if and where the pathlines left (or entered) the LV, the inflow and outflow per cardiac cycle to and from the LV can be compared 46. In accordance with the principle of mass conservation, the inflow should be equal to the outflow. Therefore, pathlines‐based estimation of LV inflow and outflow represent a very sensitive approach to quality control of 4D flow MRI velocity data. Moreover, the pathline analysis also divides the flow into four different components: direct flow, retained inflow, delayed ejection flow, and residual flow 46. The LV was segmented from the stack of short‐axis images using Segment (Medviso AB, Lund, Sweden) 49. The pathlines were computed in MATLAB (MathWorks, Natick, MA) using a four‐stage Runge‐Kutta integration scheme with quad‐linear spatiotemporal interpolation and a step length of 5 ms 44. For patient 3, the systemic right ventricle was analyzed instead of the left ventricle.

The spiral and Cartesian pathlines were also compared pairwise by visual inspection in EnSight (EnSight, CEI Inc, Apex, NC), looking for relative differences between the methods with respect to flow distribution and routes and any occurrence of abrupt or nonphysiological pathline trajectories.

The in‐vitro measurements were reconstructed with the coil sensitivity normalization filter turned off. To obtain spatially uniform noise, the images were divided by the SENSE geometry factor map. The SNR in all voxels were obtained by dividing the magnitude with the standard deviation (SD) of the uniform noise image 50. The mean and SD of the SNR in a region of interest of the phantom were then computed for the spiral and Cartesian data. The mean and SD of the velocities in the region of interest were also computed in order to compare velocity offsets in the spiral versus Cartesian data.

Additionally, a visual qualitative assessment of the velocity and magnitude images of the spiral and Cartesian 4D flow data was performed to assess the degree of aliasing artifacts (spiral foldover). The following scheme was used for grading of the amount of aliasing in the spiral data (1: no artifacts observed; 2: artifacts observed in only the phase outside the body; 3: artifacts observed in the peripheral parts of the phase or magnitude data; 4: artifacts observed in the magnitude data covering the heart and aorta; 5: artifacts observed in the phase data covering the heart).

### Statistical Analysis

Linear regression was used to test for a linear relationship and correlation between net volume flow per cardiac cycle, peak flow rate, and peak velocity in the ascending aorta (AA) and pulmonary trunk (PT) data. A least‐squares estimation was used to compute the linear regression model, AA = β·PT +α. A F‐test was used to test for a significant linear relationship, and the level of statistical significance was set at P < 0.05. Moreover, cardiac LV inflow and LV outflow from the pathline analysis were compared using the same method. The spiral and Cartesian net volume flow values from both the through plane flow and pathline analysis were also compared using linear regression. Moreover, the four pathline flow components (combined) from the spiral and Cartesian data were also compared by linear regression. To complement the regression analysis, Bland Altman plots were used to visualize the difference between the different values. Results are provided as mean ± 1 SD, unless otherwise indicated.

## RESULTS

The scan time of the spiral 4D flow MRI of the healthy volunteers was 13 ± 3 min compared to 31 ± 7 min for the Cartesian 4D flow MRI (7 healthy volunteers), corresponding to more than a two‐fold decrease in scan time.

**Figure 1:**
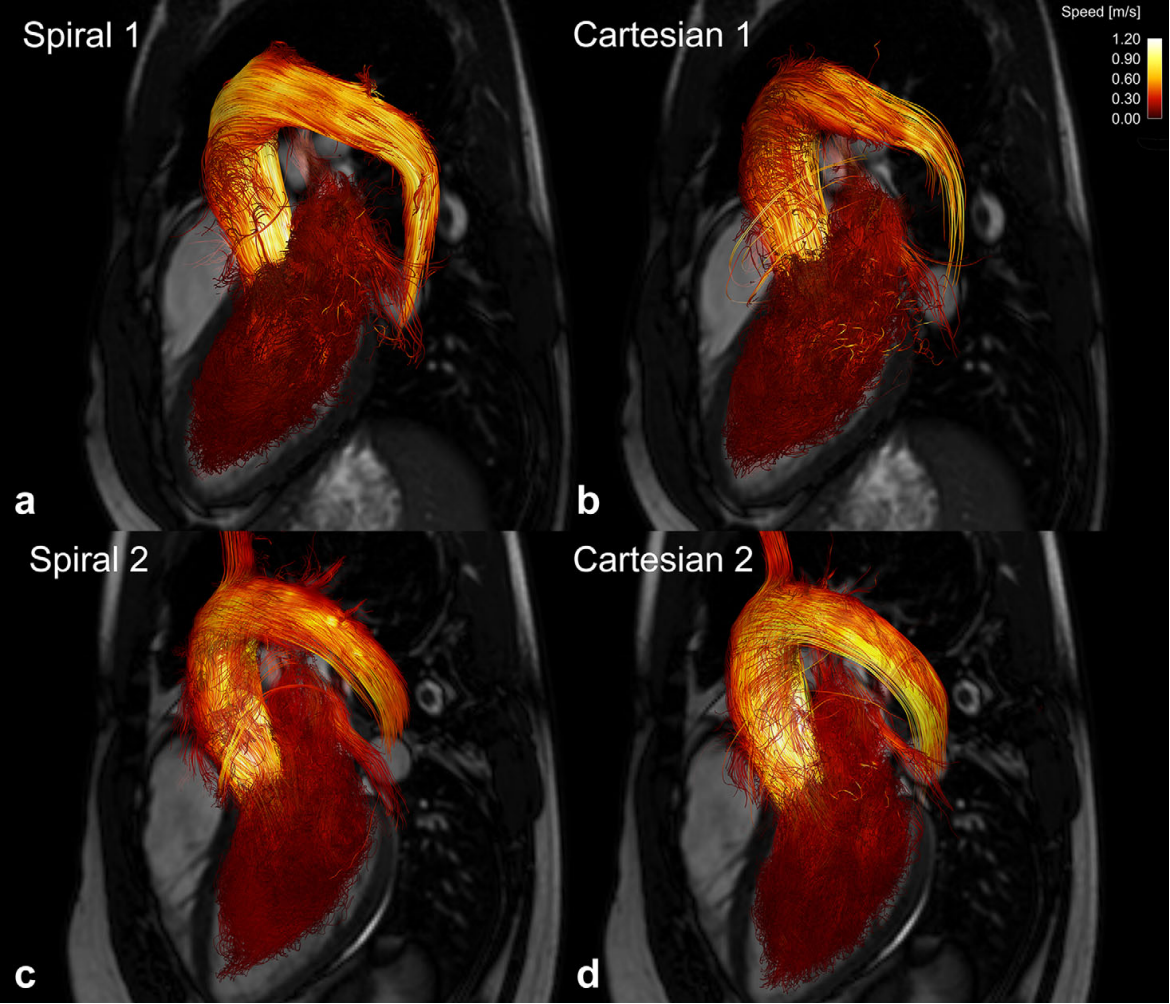
Pathlines covering complete heart cycle from two healthy volunteers from (a, c) spiral and (b, d) Cartesian 4D flow acquisition. Pathlines were released backward and forward from segmentation of left ventricle at end diastole. Three‐chamber balanced steady‐state free‐precession image is shown for orientation. Pathlines are color‐coded according to speed.

**Figure 2:**
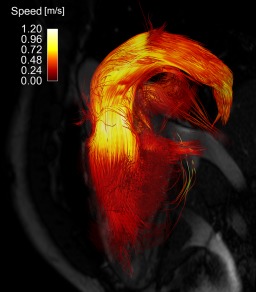
Pathlines covering complete heart cycle from spiral 4D flow acquisition of patient 3, 30‐year‐old male with repaired congenital heart disease (Senning repair for transposition of great arteries), and moderately dilated right (systemic) ventricle with mild systolic dysfunction. Pathlines were released backward and forward from segmentation of right ventricle at end diastole. Three‐chamber balanced steady‐state free‐precession image is shown for orientation. Pathlines are color‐coded according to speed.

**Figure 3:**
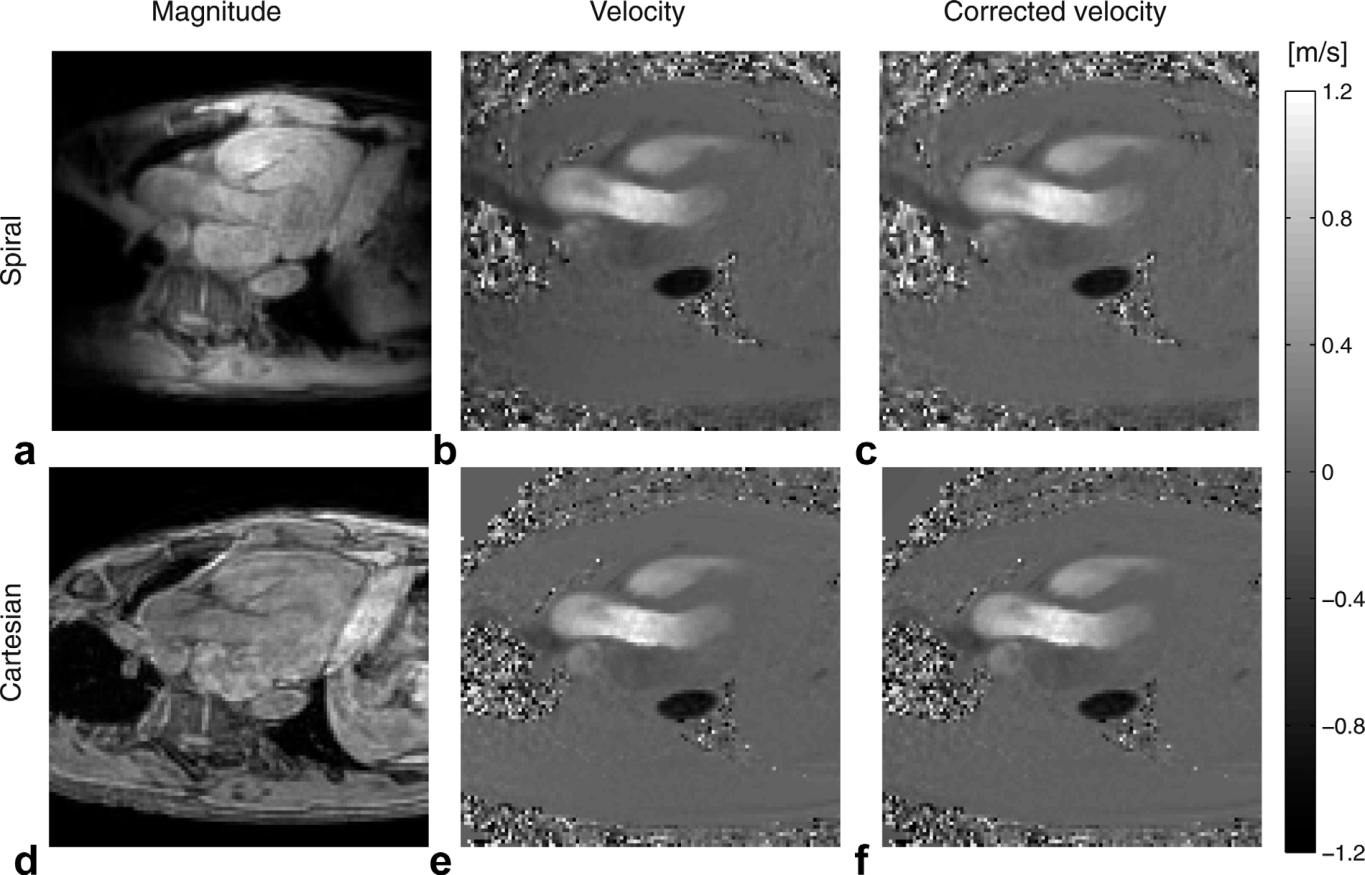
Magnitude and velocity images from spiral (a–c) and Cartesian (d–f) 4D flow images. Velocity in feet–head direction for one timeframe during systole is shown before background correction (b, e) and after background correction (c, f).

Visual inspection of the pathlines from the healthy volunteers showed no major differences between the two 4D flow acquisitions. As an example, the pathlines from the spiral and Cartesian acquisition from two of the healthy volunteers are shown in Figure 1. The pathlines from the systemic right ventricle of patient 3 are shown in Figure 2. Visual assessment of the magnitude and velocity data revealed some differences between the spiral and Cartesian 4D flow measurements (Fig. 3). In the spiral acquisition, aliasing (swirl) artifacts were present in the periphery of the FOV, but no artifacts covering the heart and aortic arch were seen. Moreover, the artifacts were seldom observed in the phase data except for outside the body. The mean aliasing score for all 10 spiral measurements was 2.3 ± 0.9; for the seven healthy volunteers it was 2.3 ± 1.1, and for the three patients it was 2.3 ± 0.6. As seen in Figure 3, phase offsets were larger in the spiral data before background correction, but no differences were seen after correction. The in‐vivo measurements indicated that the spiral data seems to be less influenced by the navigator used for respiratory gating.

**Figure 4:**
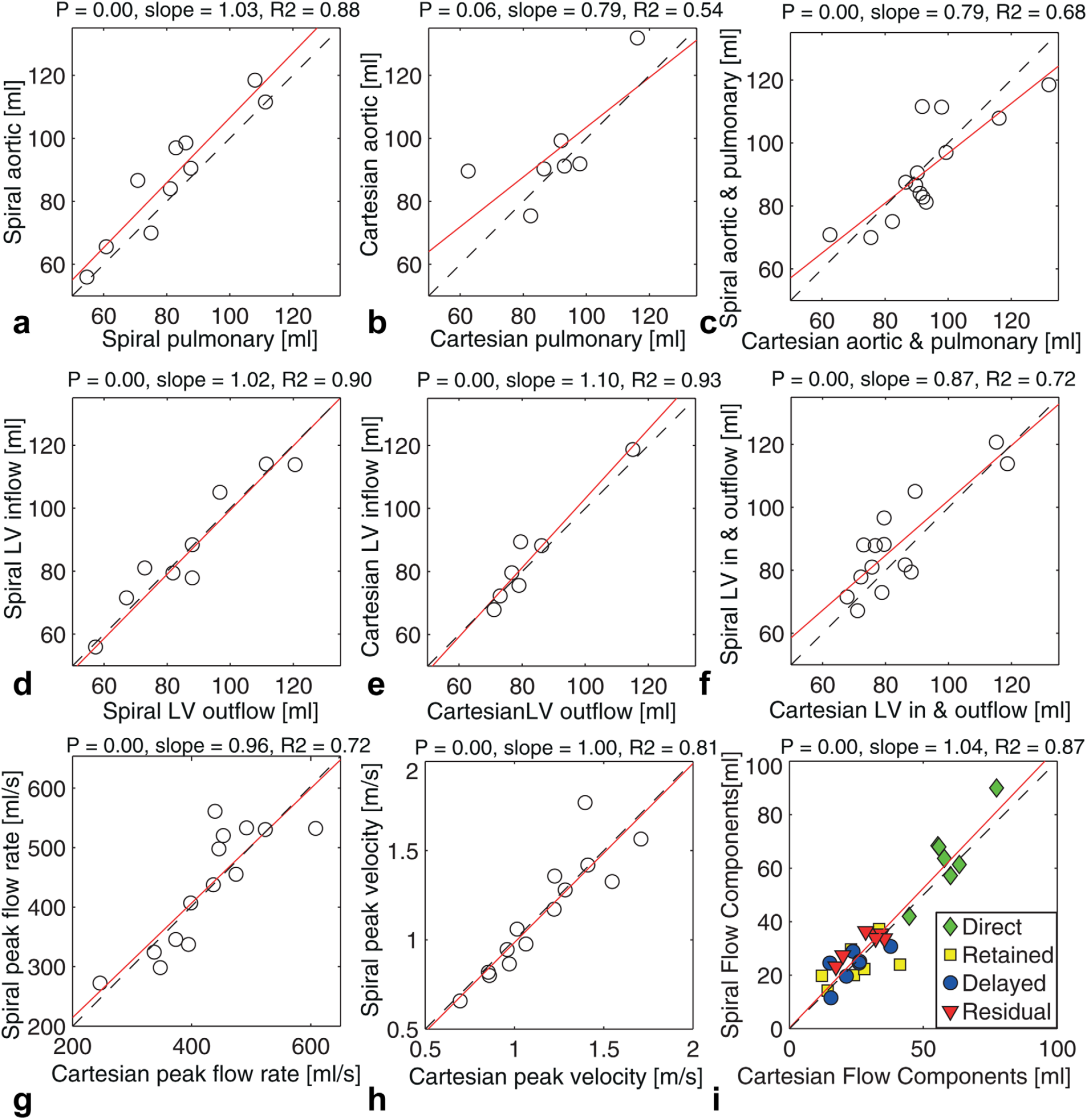
Plots of result from linear regression analysis comparing aortic and pulmonary flow for spiral (a) and Cartesian (b) data. Plots of pathline analysis left‐ventricle (LV) inflow and outflow for spiral (d) and Cartesian (e) data. Results of comparison between spiral and Cartesian flow values from aortic/pulmonary comparison (c) and pathline LV flow analysis (f) are also included. Moreover, spiral and Cartesian peak flow rate (g) and peak velocity are plotted, as well as four flow components (combined) (i). Dashed line shows identity line, and solid line shows regression line.

The results from the linear regression analysis indicate good correlation between the aortic and pulmonary flow (*P* < 0.05, slope = 1.03, R^2^ = 0.88, N = 10) for the spiral 4D flow data (Table 2, Fig. 4a). For the Cartesian data (Fig. 4b), no significant linear relationship was found (*P* = 0.06, N = 7).

**Table 2:** Results From Linear Regression Analysis

Flow Through AA Compared to PT								
y	X	Aortic Flow (ml)	Pulmonary Flow (ml)	Intercept (ml)	Slope	*P* value	R^2^	N
Spiral AA	Spiral PT	88 ± 20	82 ± 18	3.56	1.03	0.0001	0.88	10
Cartesian AA	Cartesian PT	96 ± 17	90 ± 16	24.55	0.79	0.0610	0.54	7

Pathline Analysis: LV Inflow Compared to LV Outflow								
y	X	Inflow (ml)	Outflow (ml)	Intercept (ml)	Slope	*P* value	R^2^	N
Spiral in	Spiral out	83 ± 23	84 ± 21	−2.41	1.02	0.0000	0.90	10
Cartesian in	Cartesian out	84 ± 17	83 ± 15	−6.48	1.10	0.0004	0.93	7

Spiral Compared to Cartesian: Net Volume Flow								
y	X	Spiral (ml)	Cartesian (ml)	Intercept (ml)	Slope	*P* value	R^2^	N
Spiral AA & PT	Cartesian AA & PT	91 ± 16	93 ± 16	17.63	0.79	0.0003	0.68	7 + 7
Spiral in & out	Cartesian in & out	88 ± 16	84 ± 15	14.90	0.87	0.0001	0.72	7 + 7

Peak Flow Rate								
y	X	Spiral (ml/s)	Cartesian (ml/s)	Intercept (ml/s)	Slope	*P* value	R^2^	N
Spiral AA & PT	Cartesian AA & PT	432 ± 101	426 ± 89	21.73	0.96	0.0001	0.72	7 + 7

Peak Velocity								
y	X	Spiral (m/s)	Cartesian (m/s)	Intercept (m/s)	Slope	*P* value	R^2^	N
Spiral AA & PT	Cartesian AA & PT	1.14 ± 0.32	1.16 ± 0.29	−0.02	1.00	0.0000	0.81	7 + 7

Flow Components From Pathline Analysis*								
y	X	Spiral (ml)	Cartesian (ml)	Intercept (ml)	Slope	*P* value	R^2^	
Spiral	Cartesian	36 ± 19	34 ± 17	0.28	1.04	0.0000	0.87	7·4

Linear regression model was y = intercept + slope ·X. If *P* value of slope, *P*, is < 0.05, a significant linear relationship between y and X was found. N = 7 for normal and N=10 if including patients (only spiral data). All net volume flow values are represented as mean ± one SD. *Regression analysis of flow components included all four flow components (direct, retained, delayed, and residual) in one regression. Mean flow is mean of all components.

AA, ascending aorta; LV, left ventricle; PT, pulmonary trunk; SD, standard deviation.

**Figure 5:**
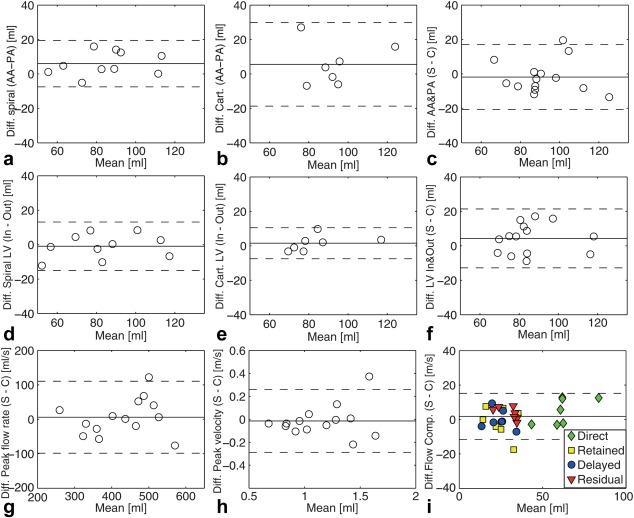
Bland‐Altman plots comparing difference between aortic and pulmonary flow for spiral data (a), with bias of 5.99 and limits of agreement ±3.49 ml, and Cartesian data (b), with a bias of 5.58 and limits of agreement ±24.27 ml. Plots comparing inflow and outflow from pathline analysis for spiral data (d), with a bias of −0.89 and limits of agreement ±14.04 ml, and Cartesian data (e), with a bias of 1.52 and limits of agreement ±8.98 ml. Plots comparing spiral and Cartesian 4D flow acquisitions: (c) Aortic and pulmonary flow, with a bias of −1.81 and limits of agreement ±18.86 ml. (f) Inflow and outflow from pathline analysis, with a bias of 4.28 and limits of agreement ±17.10 ml. (g) Aaortic and pulmonary peak flow rate, with a bias of 5.98 and limits of agreement ±104.39 ml/s. (h) Aortic and pulmonary peak velocity, with a bias of −0.01 and limits of agreement ±0.27 m/s. i) Flow components, with a bias of 1.80 and limits of agreement ±13.37 ml. Dashed lines indicate limits of agreement (±1.96 times standard deviation of mean difference).

There was good and similar correlation between the pathline‐based LV inflow and outflow for both spiral (*P* < 0.05, slope = 1.02, R^2^ = 0.90, N = 10) and Cartesian (*P* < 0.05, slope = 1.10, R^2^ = 0.93, N = 7) 4D flow data (Table 2) (Figs. 4d,e). There was a weaker linear relationship (Table 2) (Figs. 4c,f) between the spiral and Cartesian net volume flow values from the AA and PT (*P* < 0.05, slope = 0.79, R^2^ = 0.68, N = 14) and the pathline analysis (*P* < 0.05, slope = 0.87, R^2^ = 0.72, N = 14). The correlation between spiral and Cartesian peak flow rate was slightly better (*P* < 0.05, slope = 0.96, R^2^ = 0.72, N = 14) than for net volume flow. Furthermore, the correlation between spiral and Cartesian peak velocity was even better (*P* < 0.05, slope = 1.00, R^2^ = 0.81, N = 14). There was also good correlation between the pathline flow components from the spiral and Cartesian data (*P* < 0.05, slope = 1.04 R^2^ = 0.87, N = 28) (Table 2) (Fig. 4i). The mean flow components for the spiral and Cartesian data, respectively, were: direct 64 ± 14 and 59 ± 10 ml; retained 24 ± 8 and 25 ± 10 ml; delayed 24 ± 6 and 24 ± 8 ml; and retained 32 ± 5 and 29 ± 7 ml. The results of the Bland‐Altman analysis are shown in Figure 5.

The aortic and pulmonary flow, peak flow rates, and peak velocity, as well as the pathline‐based inflow and outflow from the three different patients, can be seen in Table 3. The mean difference between aortic and pulmonary flow was 6.16 ml for the patients, compared to 7.36 ml for the spiral data from the healthy volunteers. For the pathline‐based inflow and outflow, the mean difference was 5.34 ml and 5.85 ml for the patients and healthy volunteers, respectively.

**Table 3:** Patient Results

Patient	AA Flow (ml)	PT Flow (ml)	AA Peak Flow Rate (ml/s)	AA Peak Velocity (m/s)	PT Peak Flow Rate (ml/s)	PT Peak Velocity (m/s)	Inflow (ml)	Outflow (ml)	Direct Flow (ml)	Retained Inflow (ml)	Delayed Ejection (ml)	Residual Volume (ml)
1	56	55	286	1.80	296	0.80	56	57	17	39	40	87
2	66	61	354	1.34	295	0.74	46	58	36	10	22	29
3	99	86	519	1.13	529	1.26	114	111	66	48	46	75

Patient 1: 52‐year‐old female with ischemic heart disease and a heart rate of 72 beats per minute; patient 2: 19‐year‐old female with intermittent arrhythmia and a heart rate of 85 beats per minute; patient 3: 30‐year‐old male with repaired congenital heart disease (Senning repair for transposition of great arteries) and a heart rate of 54 beats per minute.

All values are represented as mean ± one SD.

AA, ascending aorta; PT, pulmonary trunk; SD, standard deviation.

The SNR obtained from the in‐vitro measurements was 323 ± 198 and 390 ± 243 for the Cartesian and spiral sequences, respectively (Table 4). The velocity offsets in the stationary phantom were −0.003 ± 0.045 and −0.070 ± 0.047 m/s for the Cartesian and spiral sequences, respectively. After background correction, the velocity offsets were negligible for both measurements (Table 4).

**Table 4:** In‐Vitro Measurements

	SNR	Uncorrected Velocity (m/s)	Corrected Velocity (m/s)
Spiral	390 ± 243	−0.07 ± 0.05	0.00 ± 0.01
Cartesian	323 ± 198	0.00 ± 0.05	0.00 ± 0.01

All values are represented as mean ± one SD.

SD, standard deviation; SNR, signal‐to‐noise ratio.

## DISCUSSION

A 4D flow MRI sequence using spiral readouts and retrospective cardiac gating was implemented. Evaluation in the heart was performed by pathline analysis and comparison of aortic and pulmonary flow. More than a 2‐fold scan time reduction was achieved compared to the Cartesian acquisition, which was accelerated using SENSE factor 2.

The comparison between the aortic and pulmonary net volume flow indicates good internal consistency for the spiral 4D flow data. The Cartesian sequence performed less well in the comparison between the aortic and pulmonary flow. A significant linear relationship may have been found if more Cartesian datasets were available, reducing the effect of outliers. The aortic flow does not include the flow to the coronaries; therefore, it should be slightly lower (less than 5% of the stroke volume) than the pulmonary flow in hearts without shunt flow. However, for both the spiral and Cartesian data, the mean aortic flow was slightly larger than the pulmonary flow, indicating that errors in the range of 10% may be present. Persistent background velocity offsets or other artifacts may have caused this discrepancy. For the Cartesian data, this discrepancy may be caused by two outliers that have a large influence in this small sample size. The pathline analysis seems to be more stable in this aspect, which may be due to less sensitivity to local errors.

The pathlines‐based comparison of the LV inflow and outflow indicate that the differences in pathline accuracy between the spiral and Cartesian acquisitions are small. However, some differences between the spiral and Cartesian net volume flow values from the AA and PT analysis and the pathline analysis were observed. Because the values compared are not measured at the same time, there may be differences in the actual flow between the spiral and Cartesian measurements. The comparison of the pathline flow components also showed some small differences between the spiral and Cartesian data; for example, the spiral direct flow was slightly larger than the Cartesian. In the pathline analysis, pathlines leaving through the myocardium are discarded; and for the spiral data slightly less pathlines were discarded, which may have caused this difference. Although the number of patients was small, no degrading in data quality was observed for the three patients compared to the healthy volunteers when comparing the aortic versus pulmonary flow and the pathline‐based inflow versus outflow. Moreover, no difference in aliasing score was seen between the patients and healthy volunteers.

We chose to perform the data quality assessment based on an in‐vivo study because this best reflects actual use of the technique. The assessment was based on volume flow and pathline analysis, which represent the two major analysis approaches in use for cardiac 4D flow MRI. A limitation of this in‐vivo approach is that the flows measured with the different acquisitions are not identical. Another limitation is the long examination time necessary for performing two 4D flow MRI measurements, resulting in a relatively small number of datasets. The long scan times also prohibited a comparison with other advanced acceleration techniques.

It is difficult to perform a fair comparison of SNR between the Cartesian and spiral acquisitions because the discrepancies between the spiral and Cartesian image reconstruction, including the gridding of spiral k‐space trajectories, can have a large influence on SNR estimates derived from the reconstructed data. The in‐vitro SNR assessment does not fully represent the in‐vivo SNR, but it gives a hint that the SNR of the two 4D flow sequences is similar. The spiral acquisition starts in the center of k‐space; thus, the signal will be higher in the center of k‐space, resulting in a gain in SNR, thanks to less T2* effects. The use of longer readouts increases the efficiency of the sequence, but this may lead to off‐resonance effects. Several methods for the correction of off‐resonance effects in spiral‐MRI have been proposed 51, 52, 53, 54, 55. Because we used relatively short spiral readouts and fat suppression, off‐resonance correction was not necessary 44. Short spiral readouts also reduce the misregistration of low and high frequencies for moving objects, which can occur when the low frequencies (center of k‐space) are acquired before the high 34, 56. Another drawback with long spiral readouts is decreased temporal resolution. By using a beat interleaved sequence and measuring only one flow‐encoding segment during a single heartbeat, the temporal resolution may be improved. However, our initial tests indicated that the velocity estimates of beat‐interleaved acquisitions were sensitive to beat‐to‐beat variations and eddy currents, resulting in velocity offsets. This may result in a decrease in data quality, which will be especially prominent in diastolic flow 44. Similar problems have been reported from spiral beat‐interleaved myocardial phase contrast 40.

In addition to the amount of SNR and smoothing, the data quality is determined by the amount of velocity offset. In spiral acquisition, the bipolar gradients are closer to the echo time, which may result in a larger sensitivity to eddy currents. In this study, we noticed slightly higher velocity offsets for the spiral data compared to Cartesian by visually studying the uncorrected velocity data. After correction, no difference in velocity offsets was observed. The correction used in this work relies on adequate signal in the static tissue in measured volume. The water‐selective pulse of the spiral sequence decreases the amount of signal from static tissue; therefore, it may decrease the quality of the offset correction.

The reconstruction of the spiral data was carried out offline, and the reconstruction time is similar to the time required for the Cartesian data. Reconstruction on the scanner should be possible. However, the current implementation might need some memory optimization in order to be able to handle data that has been acquired with a large number of coil channels and large FOV.

A combination of spiral readouts and parallel imaging may be used to further accelerate 4D flow measurements. Although the reconstruction of spiral SENSE data has proved to be computationally burdensome 57, 58, the GRAPPA algorithm 18 has been successfully adapted to spiral imaging without causing unreasonable reconstruction times 59. Furthermore, the combination of variable density spiral readouts and compressed sensing parallel imaging has shown promising results for functional MRI 60.

Retrospective cardiac gating allows for coverage of the complete cardiac cycle and, for example, the assessment of the late diastolic filling phase and improved pathline analysis. Despite some obvious advantages of retrospective gating compared to prospective gating, retrospective gated 4D flow MRI is still unavailable on many MRI systems. This has resulted in a relatively small number of cardiac 4D flow MRI studies as compared to vascular studies for which diastolic blood flow may be less important. In this study, we have developed a retrospectively gated spiral 4D flow MRI sequence and evaluate it for the assessment of cardiac blood flow. We hope that this study also motivates others to improve the availability of retrospectively gated 4D flow MRI.

## CONCLUSION

Retrospectively gated spiral cardiac 4D flow MRI permits more than two‐fold reduction in scan time compared to conventional Cartesian 4D flow MRI (SENSE factor 2), while maintaining similar data quality. The time‐savings offered by spiral trajectories provides a necessary step in bringing 4D flow MRI closer to routine clinical use.
